# Clinical application of confocal laser endomicroscopy in the diagnosis of malignant pleural effusion

**DOI:** 10.3389/fonc.2025.1666259

**Published:** 2026-01-12

**Authors:** Shenglan Ye, Mingli Yuan, Cheng Song, Zhen Yang, Yi Hu, Jixiang Ni

**Affiliations:** Key Laboratory for Molecular Diagnosis of Hubei Province, The Central Hospital of Wuhan, Tongji Medical College, Huazhong University of Science and Technology, Department of Respiratory and Critical Care Medicine, Wuhan, Hubei, China

**Keywords:** probe-based confocal laser endomicroscopy (pCLE), medical thoracoscopy (MT), malignant pleural effusion (MPE), diagnostic value, pleural effusion

## Abstract

**Objective:**

Probe-based confocal laser endomicroscopy (pCLE) is an innovative *in vivo* microscopic imaging technique that enables real-time visualization of tissue cytology during endoscopic examinations. This study aimed to evaluate the clinical utility of pCLE in diagnosing MPE.

**Methods:**

A total of 41 patients with pleural effusion (PE) who underwent pCLE examination were enrolled in this prospective study. The diagnostic performance of pCLE was assessed by calculating sensitivity (SEN), specificity (SPE), positive predictive value (PPV), negative predictive value (NPV), and accuracy (ACC), using histopathological results as the reference standard. Additionally, the safety profile of the pCLE procedure was evaluated.

**Results:**

Among the 41 patients, histopathological analysis confirmed MPE in 31 patients and benign pleural effusion (BPE) in 10 patients. Of the 31 MPE cases, 26 were lung adenocarcinoma (LUAD), 2 were lung squamous cell carcinoma (LSCC), 1 was large cell lung cancer (LCLC, a subtype of non-small cell lung cancer [NSCLC]), 1 was pleural malignant mesothelioma (PMM), and 1 was pleural metastasis from other tumors. Compared with histopathology, pCLE demonstrated a sensitivity of 96.77%, specificity of 80%, accuracy of 92.68%, PPV of 93.75%, and NPV of 88.89%. There was substantial agreement between pCLE and histopathological findings (kappa = 0.79, p < 0.001). Furthermore, compared to conventional thoracoscopy, pCLE facilitated targeted biopsy sampling and improved detection of malignant lesions. No adverse events related to pCLE were observed, indicating its favorable safety profile.

**Conclusions:**

Our study demonstrates that pCLE is a valuable tool for enhancing the diagnosis of MPE, with high concordance with histopathological findings (kappa = 0.79, p < 0.001) and significant guidance for thoracoscopic pleural biopsies. The diagnostic sensitivity, specificity, accuracy, positive predictive value, and negative predictive value of pCLE were 96.77%, 80%, 92.68%, 93.75%, and 88.89%, respectively, when compared with histopathology. These results indicate that pCLE can effectively differentiate between benign and malignant pleural effusion in patients with undetermined etiology and serves as a robust complementary method to existing diagnostic approaches. However, the small sample size of this study limits the generalizability of the findings, and further large-scale, multicenter clinical trials are needed to validate these results.

## 1 Introduction

Pleural effusion, which arises from a variety of underlying diseases, is associated with a high incidence in clinical practice. Early detection and accurate identification of MPE are essential for the timely diagnosis, appropriate treatment, and favorable prognosis of affected patients (1–3). With ongoing advancements in medical diagnostic technologies, thoracoscopy has increasingly become the preferred modality for pleural effusion evaluation and pathological sampling due to its high safety profile and minimally invasive characteristics. It demonstrates particular advantages in the diagnosis of undiagnosed or unexplained pleural effusions (4–7). Nevertheless, accumulating evidence suggests that thoracoscopy exhibits limited efficacy in distinguishing benign from MPE, with an overall diagnostic accuracy of approximately 68.9%, indicating notable constraints (8).

Probe-based confocal laser endomicroscopy (pCLE) is a novel imaging technique, enables non-invasive, real-time histological assessment at the cellular level *in vivo*, offering dynamic, high-resolution imaging capabilities (9, 10), thereby addressing the limitations of low diagnostic accuracy associated with visual inspection during conventional thoracoscopy. The pCLE probe can be introduced into the pleural cavity through the working channel of an endoscope, allowing for real-time cellular-level imaging during procedures. This technology represents a significant advancement in enabling real-time, *in vivo* histological evaluation at the microscopic level (11). Rapid on-site cytological analysis using pCLE is highly time-efficient and provides reliable diagnostic support for clinical decision-making. Currently, pCLE has been widely adopted in the field of gastroenterology, significantly improving the diagnostic accuracy of gastrointestinal lesions (12–14). Moreover, pCLE has demonstrated considerable diagnostic value in various other disease contexts (15–18).

This study aims to investigate the potential application of pCLE in the detection of MPE, with the objective of overcoming the limitations of conventional thoracoscopy in terms of spatial resolution and reducing unnecessary invasive interventions by guiding targeted biopsies. The findings may offer a novel and effective clinical approach for the diagnosis and management of respiratory diseases. The following sections detail the methodology and results of this investigation.

## 2 Methods

### 2.1 Clinical information

This prospective study included 41 patients with pleural effusion admitted to our hospital from April 2022 to October 2024. The gender, age and pathological diagnosis distribution of these patients are detailed in **Table 1**. The content of the informed consent form was approved by our Ethics Committee, and all patients received follow-up treatment in our hospital. Study inclusion criteria included (1) adults (≥18 years of age), (2) no contraindications to medical thoracoscopy, and (3) failure to determine the etiology of the pleural effusion by conventional pleural fluid examination. Exclusion criteria included (1) patients who were deemed to have poor compliance and were unsuitable for participation in the study by the attending physician; (2) pregnant or breastfeeding women; (3) allergy to fluorescein sodium injection; (4) coagulation disorders; (5) known allergies, a history of asthma, or hepatic/renal failure as contraindications for fluorescein sodium injection; (6) acute respiratory failure accompanied by hypercapnia; (7) high airway obstruction; (8) patients with poor oxygenation during the examination; (9) untreated fatal arrhythmias; and (10) recent history of myocardial infarction; (11) severe cardiopulmonary insufficiency, such as severe heart failure, severe chronic obstructive pulmonary disease, or pulmonary hypertension, which could not tolerate the respiratory and circulatory changes during thoracoscopy and might lead to serious cardiopulmonary complications; (12) severe cardiovascular diseases, such as severe acute myocardial infarction within the past 3 months, recent severe angina attacks, biventricular failure with significant cardiac enlargement and heart function grade III or above, or severe ventricular arrhythmias; (13) severe liver or kidney dysfunction, which could not normally metabolize anesthetic drugs and stress responses during surgery, potentially leading to drug accumulation and further deterioration of organ function; (14) severe chest wall deformities, which might affect the operating space and visual field of thoracoscopy, increasing the difficulty and risk of surgery; (15) persistent uncontrollable cough, which would prevent the examination from being conducted; (16) extreme weakness or poor general condition, which would not tolerate thoracoscopy; (17) allergy to anesthetic drugs, which might trigger severe allergic reactions and endanger life.

**Table 1:** Basic information and pathological diagnosis of 41 patients in the research groupItems.

Patient characteristics	Value/Number (%)
Age (years) (mean ± standard deviation)	59.07 ± 11.68
Sex (male/female)	25/16
Pathological diagnosis	
Benign lesion	
Inflammatory infiltration, n (%)	8 (19.51%)
Granulomatous inflammation, n (%)	2 (4.88%)
Malignant lesion	
Lung adenocarcinoma, n (%)	26 (63.41%)
Squamous lung cancer, n (%)	2 (4.88%)
Large cell lung cancer, n (%)	1 (2.43%)
Pleural malignant mesothelioma, n (%)	1 (2.43%)
Pleural metastases from other tumors, n (%)	1 (2.43%)

### 2.2 Endoscopic procedures and image acquisition

Prior to the examination, an allergy test was conducted on all patients using 0.2 mL fluorescein sodium (NaFl; Alcon Laboratories, Inc; 5mL: 0.5g (10%)). Patients who showed no allergic reaction after observation for about 15 min observation period were eligible for the subsequent examination (19, 20). The thoracoscopic procedure was carried out in accordance with the standard operating protocols. Patients were positioned laterally with the contralateral upper limb elevated. The incision site was marked at the seventh intercostal space in the anterior axillary line, with the location of the thoracoscopic channel determined by imaging or bedside B-mode ultrasound. The marked incision area was sterilized. Following routine induction of anesthesia and sedation, analgesic treatment was administered, and thoracoscopy was performed. A 2.0-cm incision was made along the intercostal space, and the muscularis propria was reached through blunt separation. A trocar was inserted vertically into the thoracic cavity from the incision, and the core was withdrawn to allow air entry into the thoracic cavity. Subsequently, a flexible thoracoscope was inserted into the trocar and into the pleural cavity to observe the color and morphologic structure of the parietal and visceral pleura.

**Figure 1:**
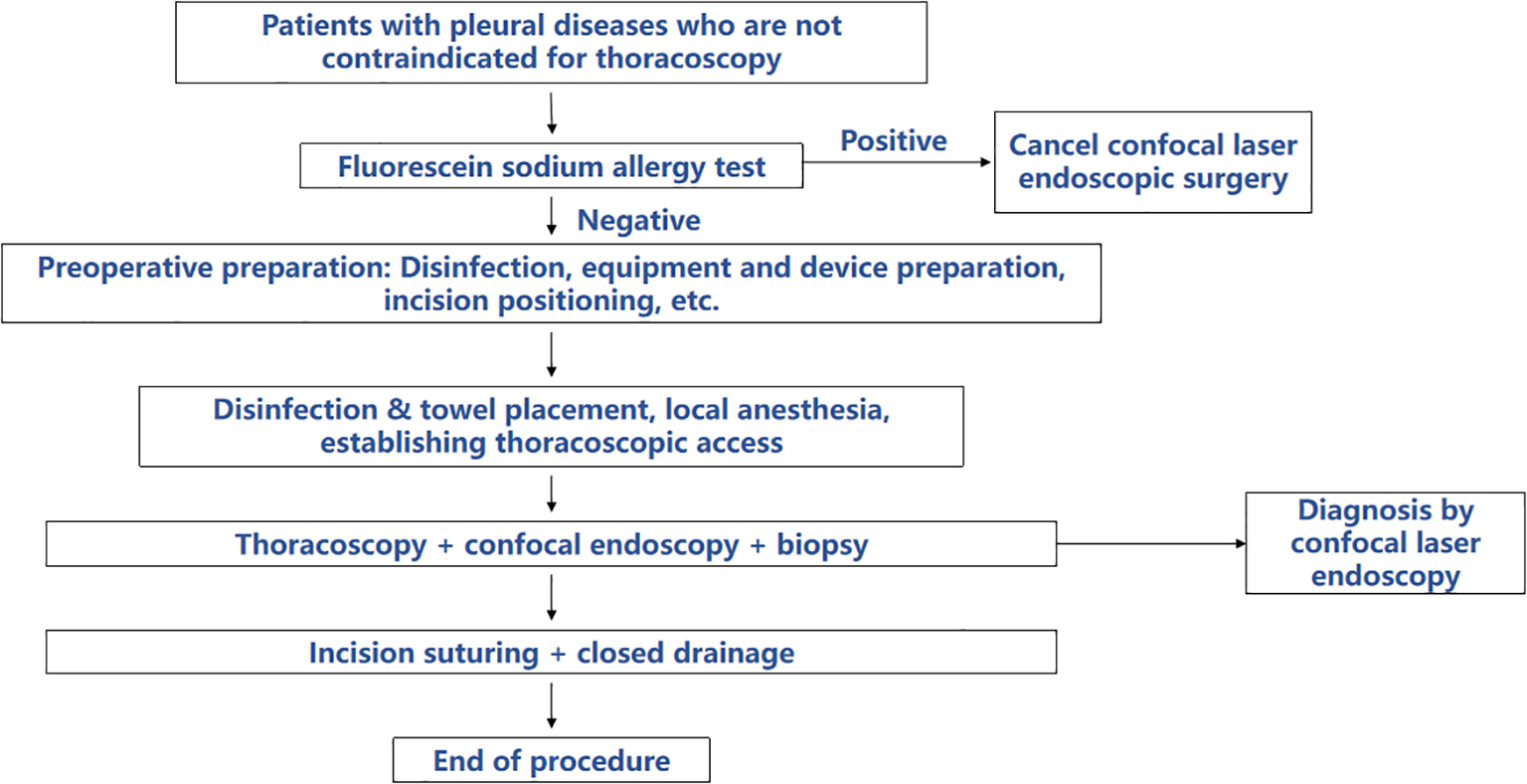
All patients underwent fluorescein sodium allergy testing; those with negative results proceeded to MT and pCLE. pCLE images were interpreted blindly, followed by targeted biopsy. Histopathology was used as the gold standard to assess pCLE performance. Discordant cases were reviewed by a third expert.

After the completion of standard thoracoscopy, CLE was performed using a probe-type laser confocal endomicroscope (BIOPSEE^®^, Viestar Medical Technology Co., Ltd.). Intravenous injection of 2mL of 10% NaFl was administered before image acquisition. The pCLE probe was inserted into the pleural cavity via the thoracoscopic working channel and positioned close to the pleura for confocal laser examination (Laser Wavelength: 488nm, Image Acquisition Speed: 4–6 frames per second, Field of View: 240μm×240μm, Resolution: 1μm). Subsequently, pCLE scanning was performed on the suspicious areas, and the corresponding image information was captured. Following the completion of the confocal microscopy, the confocal microprobe was withdrawn, and the cryoprobe and biopsy forceps were inserted to obtain several tissue samples from the confocal examination site for pathological examination. During the procedure, an appropriate amount of yellow pleural effusion was retained and sampled for examination. Finally, a closed drainage tube was left in place at the incision site. The specific operation steps are shown in **Figure 1**.

### 2.3 CLE and histopathological image analysis

Biopsy specimens were fixed in 10% neutral buffered formalin, embedded in paraffin, sectioned (4 μm), and stained with hematoxylin-eosin (HE). Meanwhile, intraoperative probe-based confocal laser endomicroscopy (pCLE) images were initially evaluated by an experienced thoracic surgeon to guide intraoperative biopsy sampling and preliminary diagnosis. Subsequently, a consulting pathologist who was not involved in the histopathological diagnosis of the specimens further analyzed and validated the pCLE images against the final pathological results. Notably, the pathologists who made the histopathological diagnoses did not assess the pCLE images intraoperatively; instead, they provided timely post-operative pathological reports. To ensure the relative independence between pathological results and pCLE diagnoses, the pathologists responsible for the histopathological diagnoses were excluded from the comparative analysis of pathological results and pCLE imaging findings. All HE sections were reviewed by two senior pathologists (with >10 years of pathology experience) who were blinded to the pCLE results. For cases with ambiguous results (e.g., suspected malignancy with insufficient tissue), immunohistochemical staining (e.g., thyroid transcription factor-1 [TTF-1] for lung adenocarcinoma, p40 for squamous cell carcinoma, calretinin for mesothelioma) was performed to confirm the diagnosis. For false-positive/negative pCLE cases, histopathological slides were re-reviewed by a third senior pathologist to verify the result.

### 2.4 Statistical analysis

Statistical analysis was performed using GraphPad Software. Sensitivity (SEN), specificity (SPE), accuracy (ACC), positive predictive value (PPV), and negative predictive value (NPV) were calculated based on the pathological results that were ultimately made available. All measurement data were expressed as mean ± standard deviation, and tested with independent t-test; count data were expressed as frequency and corresponding percentage [(%)], with two decimal places retained, and χ2 test with continuity correction was used for testing. The diagnostic efficacy of CLE was verified by descriptive analysis of the area under curve of receiver operating characteristic curve, SEN, SPE, PPV, and NPV, using histopathology as the gold standard for the diagnosis of benign and malignant pleural effusion diseases. Descriptive analysis of adverse events was also conducted.

## 3 Results

### 3.1 Patient characteristics and pathologic diagnosis

Forty-one patients (25 males and 16 females) were included in the study between April 2022 and October 2024. They had a mean age of 59.07 years, ranging from 41 to 86 years. All patients underwent thoracoscopy and confocal endoscopy sequentially. The final pathologic diagnosis of benign lesions was made in 10 of the 41 patients (8 inflammatory infiltrates and 2 granulomatous inflammation), while the remaining 31 were diagnosed to have malignant lesions. Among them, 26 patients were diagnosed to have lung adenocarcinoma, accounting for 63.41%; 2 lung squamous carcinoma, accounting for 4.88%; 1 large cell lung cancer, 1 pleural malignant mesothelioma and 1 pleural metastasis of other tumors, each accounting for 2.43%. None of the patients had endoscopic complications or adverse reaction events to sodium fluorescein during the examination. Specific data are shown in **Table 1**.

### 3.2 Diagnostic effectiveness analysis

After the completion of confocal endoscopy for all patients, image analysis was conducted, and the confocal diagnostic results were determined prior to the issuance of the pathology reports. The study by Bonhomme et al. (21) on pleural correlation was used as a reference for the diagnosis of confocal endoscopic images. A 2 × 2 table was constructed for χ2 test to calculate the diagnostic concordance between the confocal and the final pathological diagnostic results. The results demonstrated that the diagnostic results from the pCLE were highly concordant with the gold standard histopathologic findings (kappa = 0.79, *p* < 0.001) (**Table 2**).

**Table 2:** Comparison of pCLE diagnosis results and Pathology gold standard results.

pCLE examination	Pathology gold standard		Total
	Positive	Negative	
Positive	30	2	32
Negative	1	8	9
Total	31	10	41
Kappa	0.79		
*P-*value	<0.001		

pCLE diagnostic efficacy was statistically analyzed against the final histopathological diagnoses (SEN, SPE, ACC, PPV, and NPV), and 95% confidence intervals were calculated. Analysis of the statistical results showed that the SEN, SPE, ACC, PPV, and NPV of the pCLE examination were 96.77%, 80%, 92.68%, 93.75% and 88.89%, respectively, when compared with the histopathologic findings (**Table 3**).

**Table 3:** Agreement between pCLE and histopathological diagnosis (2×2 contingency table).

Diagnostic metrics'	Specific values of diagnostic efficacy	
	(%)	95% Confidence Interval (CI)
Sensitivity	96.77	81.49-99.83
Specificity	80	44.22-96.46
Accuracy	92.68	87.41-97.96
Positive predictive value	93.75	77.78-98.91
Negative predictive value	88.89	50.67-99.42

### 3.3 Analysis of confocal images in benign pleural lesions

**Figure 2:**
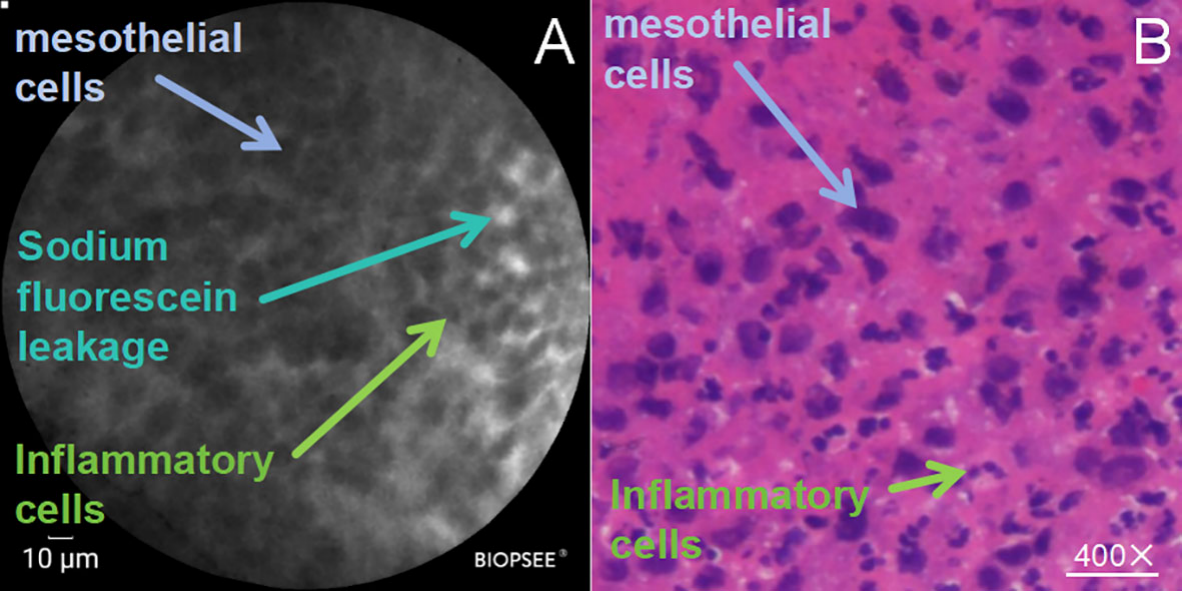
pCLE examination of inflammatory pleura; **(A)** pCLE microscopic presentation of inflammatory pleura; **(B)** Histopathology (HE staining,×200); mesothelial cells (blue arrows), inflammatory cells (green arrows), and sodium fluorescein leakage (lake green arrow).

Of the 41 patients included in the study, 9 patients demonstrated inflammatory changes on pCLE microscopy, of whom 1 was subsequently confirmed to have a malignant pleural lesion via histopathology. We compared the pCLE microscopic presentation of inflammatory pleural lesions with histopathology (**Figure 2**). Similar to the histopathological results, the inflammatory pleura showed a large amount of mesothelial cells (blue arrows) in a chia-seed-like scattered distribution on pCLE microscopy, and inflammatory cells were scattered between mesothelial cells, which were smaller than mesothelial cells (green arrows). Histopathologically, a large number of inflammatory cells infiltrating the interstitial spaces of pleural tissues was seen. Sodium fluorescein leakage was also observed on pCLE microscopy (lake green arrow).

### 3.4 Analysis of confocal images in malignant pleural lesions

**Figure 3:**
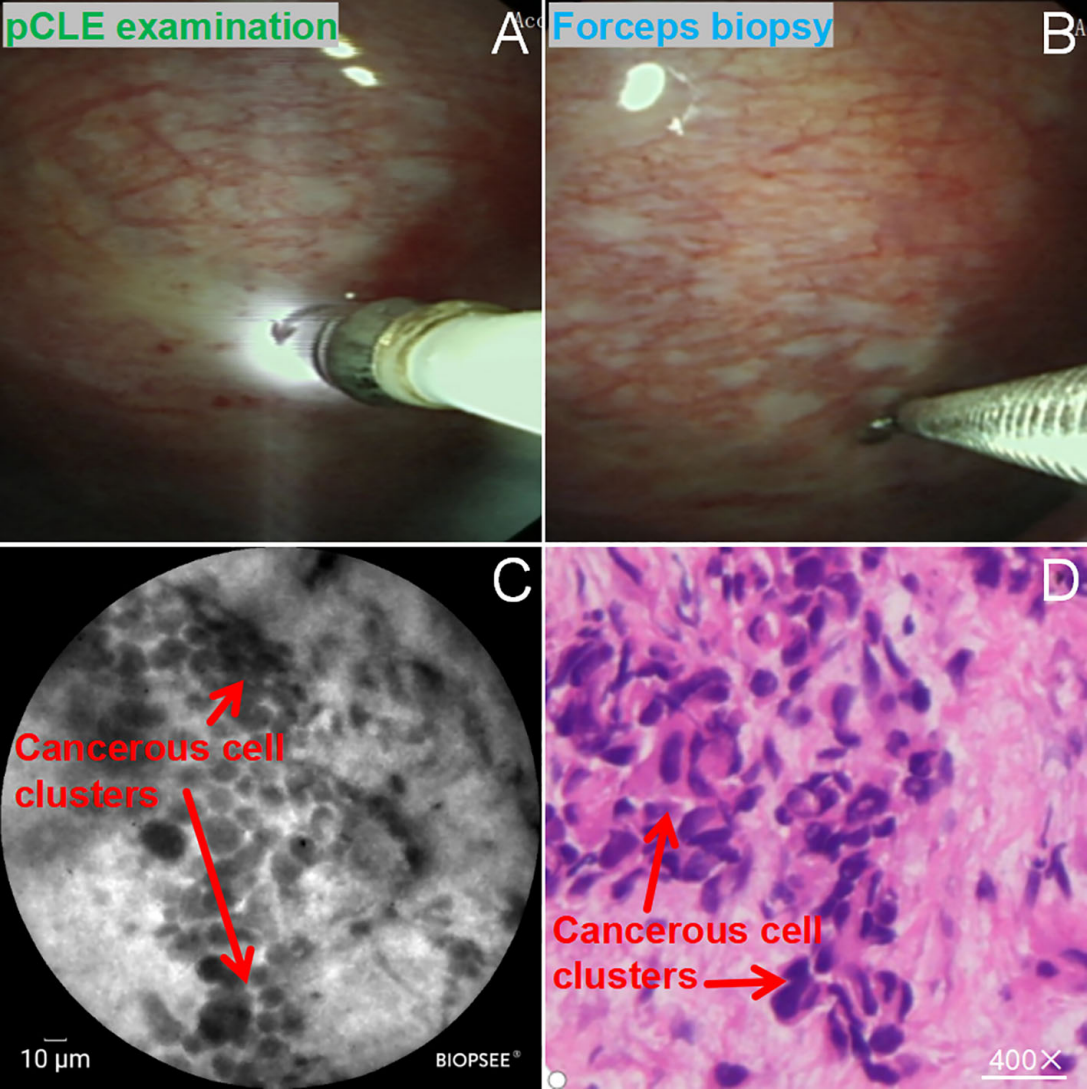
pCLE examination of malignant pleura; **(A)** Thoracoscopic confocal endoscopic examination of pleural surface; **(B)** confocal microscopic positioning-guided pleural biopsy; **(C)** pCLE microscopy showing dark black granular xenomorphic cells in clusters (red arrows) **(D)** Histopathology (HE staining, ×200) showing malignant cell infiltration (red arrows).

In patients with early pleural lesions, there were no obvious lesion features on the pleural surface under thoracoscopy (**Figure 3A**), and scanning of the surface using CLE revealed a large number of heterogeneous dark black granular cell clusters under the microscope (**Figure 3C**); pleural biopsy was performed on the designated site of the pleura using biopsy forceps (**Figure 3B**), and the final histopathologic structure obtained showed a large number of malignant cell infiltrates (**Figure 3D**). Malignant pleura appeared on CLE as dark black particles (red arrows), which varied in size and shape and were often aggregated.

**Figure 4:**
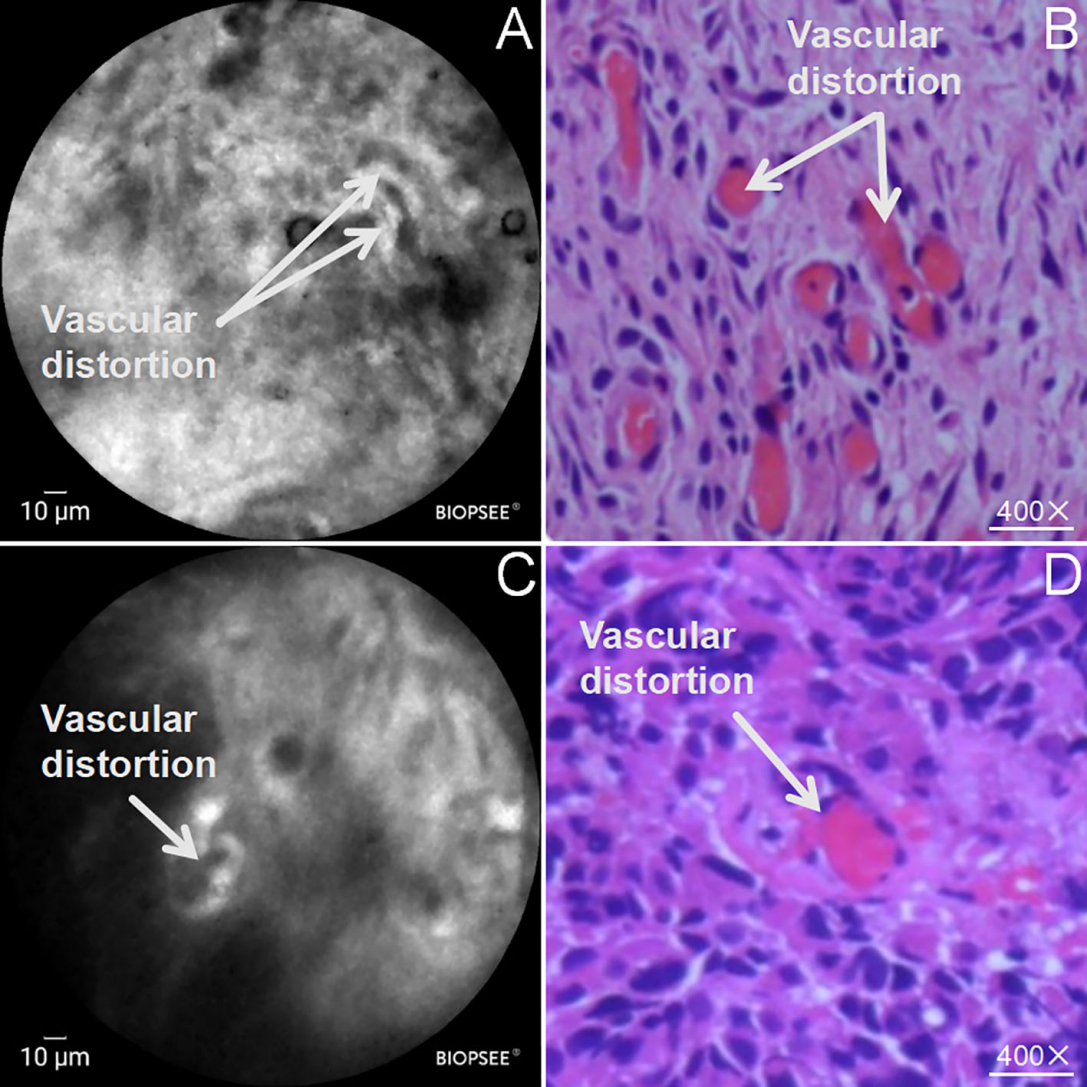
Abnormal glandular epithelial structures in malignant pleura examined by pCLE; **(A, C)** abnormal glandular epithelial structures (yellow arrows) and tumor cells distributed in clusters (red arrows) on the surface of the pleura on pCLE microscopy; **(B, D)** abnormal glandular epithelial structures (yellow arrows) and tumor cell infiltration (red arrows) in the Histopathology (HE staining, ×200).

**Figure 5:**
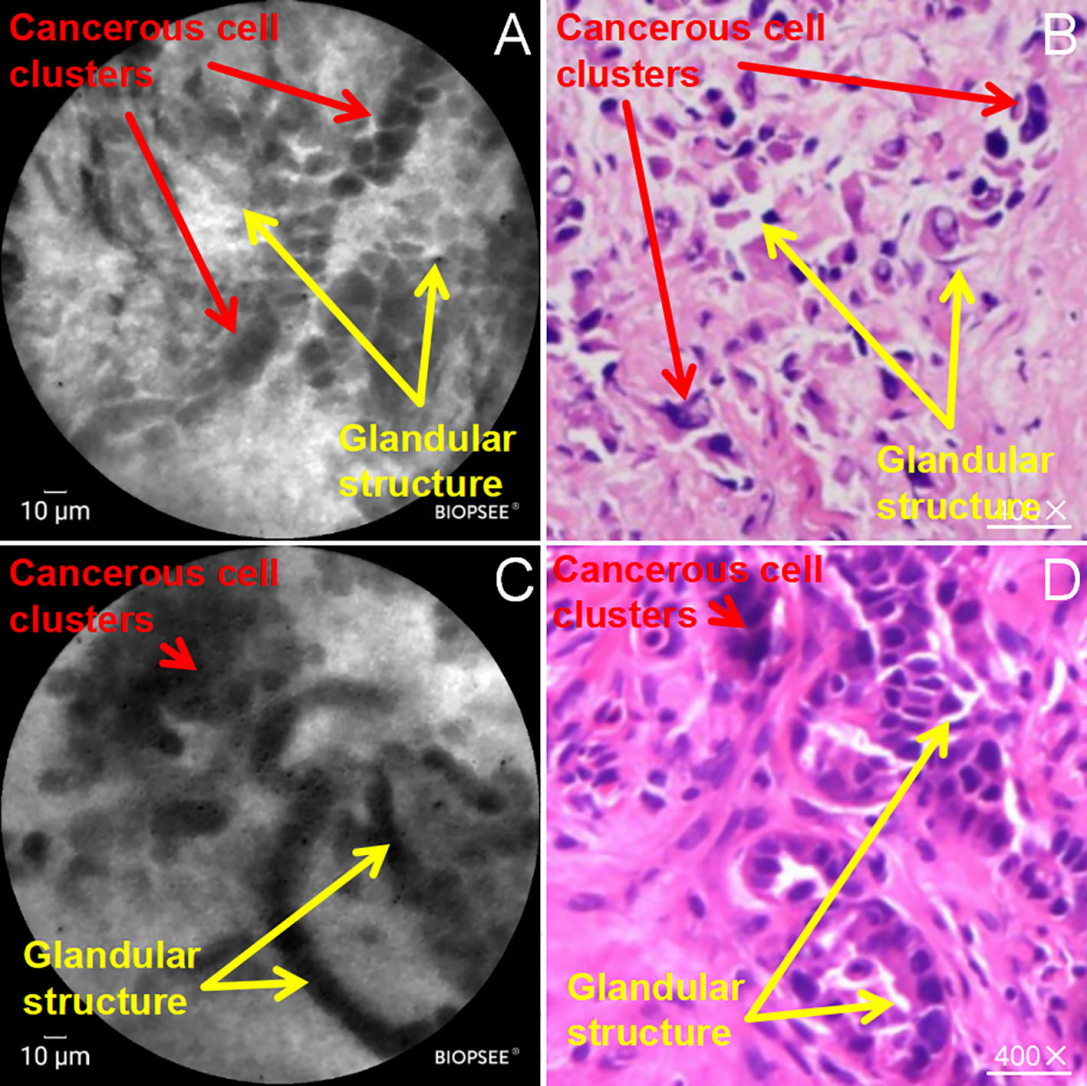
Abnormal vascular structures in malignant pleura examined by pCLE; **(A, C)** pCLE microscopy of the pleural surface showing twisted vascular structures (white arrows); **(B, D)** Histopathology (HE staining, ×100) showing twisted vascular structures (white arrows).

During the detailed analysis of the structure of the malignant pleural pCLE images, it was found that in addition to the above characteristics of the malignant tumor cells in a mass distribution, abnormal glandular epithelial structures were also observed on the pleural surface under the pCLE microscope, and the glandular cells were arranged to form glands or showed a nested or tubular distribution, accompanied by the distribution of heterogeneous black granular cells (**Figures 4A, C**); in correspondence with the pCLE microscopic structures, the final biopsy histopathological results showed that the pleural tissue showed abnormal glandular tubular structures with tumor cell infiltration (**Figures 4B, D**). In addition, abnormal vascular structures were observed on the surface of the malignant pleura on pCLE microscopy, with distorted shapes and a spiral pattern in some of the abnormal vessels (**Figure 5**). These findings were confirmed via histopathologic results, which demonstrated vascular distortion in pleural tissue in histopathologic sections. The pCLE microscopic structures were highly consistent with the final histopathological section observations.

## 4 Discussion

In this study, we recorded and detailed intraoperative pCLE images of patients with pleural effusion, which were carefully reviewed by an experienced endoscopist after the procedure. The confocal diagnosis was confirmed after redacting all basic patient information and was statistically analyzed against the final pathologic diagnosis. In this study, we found that the pCLE diagnostic results had high concordance with the final histopathologic diagnostic results, demonstrating that the pCLE examination can be used as an effective tool in diagnosing benign and malignant pleural effusions.

Currently, medical thoracoscopy is regarded as the most effective method for diagnosing and treating pleural diseases. A meta-analysis reported the sensitivity and specificity of medical thoracoscopy for diagnosing benign and malignant pleural effusions were 91% and 100%, respectively. However, it is worth noting that the studies included in this meta-analysis compared pathologic biopsies of pleura with obvious thoracoscopic presentations with thoracoscopic diagnoses (22). However, there is a high probability of missed diagnosis of some early-stage localized malignant lesions that are difficult to detect with the naked eye in thoracoscopy. One pivotal study highlighted this issue, showing that 15.4% of patients with non-specific pleural lesions diagnosed via thoracoscopy were diagnosed with malignant tumors during postoperative follow-up (23, 24). This highlights the potential for missed diagnosis of patients with unremarkable early pleural lesions by direct-view thoracoscopy and biopsy, which may have been overlooked in general studies because of limited follow-up duration. We also found an interesting phenomenon related to this during this study: before the confocal endoscopy of the pleura was started in this hospital, a low percentage of patients with unexplained pleural effusion were diagnosed as malignancy, and this percentage increased significantly after this study was performed. There is insufficient evidence to directly attribute this increase in the detection rate to the use of pCLE. Increased prevalence of MPE, changes in patient populations’ willingness to seek care, and improved physician practices all may have contributed to the higher detection rate of MPE, and follow-up studies are needed to confirm this. Currently, patients with advanced malignant pleural lesions in clinical practice have a high rate of postoperative recurrence and a low cure rate; early detection of malignant pleural lesions and appropriate treatment can benefit patients. However, early detection of malignant pleural lesions is the current hotspot and challenge in thoracic surgery and respiratory medicine.

Early-stage malignant diseases of the respiratory system have a certain degree of crypticism, but may cause serious damage to the respiratory function and peripheral important tissues and organs with the disease progression. Therefore, early diagnosis is a prerequisite for achieving a satisfactory prognosis (25–27). A variety of respiratory tumors can induce pleural effusion, and the presence of pleural effusion is one of the common signs of progression of respiratory tumors (28, 29). At the same time, some pleural metastatic lesions such as breast cancer and gastric cancer can also induce pleural effusion; therefore, differentiation between benign and malignant pleural effusions is important for achieving precision treatment as well as improving patients’ prognosis (30). The pCLE is a novel imaging technology that enables noninvasive histologic examination at the *in vivo* level to obtain high-resolution real-time dynamic images (31). The pCLE technology began to be used in clinical applications in 2000 (32). In 2007, foreign researchers began to explore its application to the respiratory system (33). In 2017, the pCLE technology was first introduced to China for exploration of image features of lung cancer (34). At present, application of pCLE technology in the digestive system is the most mature, and a CLE atlas related to digestive system diseases has been established, which is widely used to assist diagnosis of digestive system diseases, while its application to the respiratory system is still at the clinical study stage. In recent years, several scholars applied the CLE technology for real-time *in vivo* exploration of airways and lung tissues to describe the confocal microscopic normal-tissue structure and CLE imaging features of different types of respiratory diseases, and found that CLE had satisfactory application prospects for diagnostic assistance in determining respiratory disease type, assessment of disease severity, and regular postoperative follow-up (20, 35). During this study, we found that in some patients with early lesions, there were no obvious abnormal changes in the pleura by naked eye during thoracoscopy, with locally smooth regions without bulging or unevenness, and without obvious nodular hyperplasia, congested vesicles, or necrosis. Conventional endoscopy can only blindly remove biopsy samples from multiple points on the pleural surface for histopathologic examination. In this study, the pleural surface was scanned and observed by inserting the pCLE probe through the working channel of the thoracoscope, and abnormal black heterogeneous granular cells were distributed in clusters; by performing targeted biopsy after confocal microscopic observation, the final histopathology confirmed the diagnosis of lung adenocarcinoma. Therefore, we concluded that pCLE has great technical advantages in the diagnosis and treatment of patients with early pleural lesions. On one hand, it reduces the risk of hemopneumothorax caused by expanding the area of biopsy due to the absence of obvious abnormality by naked-eye observation in thoracoscopy. On the other hand, this new technology increases the detection rate of patients with malignant pleural effusion, which largely solves the current difficulty of diagnosing early malignant lesions.

Beyond its diagnostic advantages, the cost-effectiveness of pCLE technology merits attention. The costs associated with pCLE application primarily include equipment purchase, probe usage, and NaFl administration. Although the initial investment in pCLE equipment is relatively high, the technology significantly improves diagnostic accuracy and efficiency. It reduces the need for unnecessary biopsies and surgeries, enhances the detection rate of malignant lesions, and lowers the risk of advanced metastasis of these lesions. Thus, from a long-term perspective, pCLE demonstrates considerable cost-effectiveness. Compared with traditional pathological methods, pCLE offers several distinct advantages: it provides real-time, non-invasive diagnostic information intraoperatively, thereby minimizing tissue damage caused by repeated biopsies and reducing field contamination. Additionally, it decreases the time and costs associated with postoperative pathological examinations. Similar benefits have been observed in other surgical fields, such as gastrointestinal surgery. A meta-analysis on the diagnostic efficacy of confocal endoscopy in gastric cancer (36) showed that pCLE achieved a pooled sensitivity of 87.9%, specificity of 96.5%, and accuracy of 94.7% in detecting gastric cancer. These findings highlight the high-precision diagnostic performance of pCLE for gastric cancer *in vivo*, further supporting its potential cost-effectiveness in clinical practice.

For pleural effusion diseases, a small number of small-scale studies have shown preliminary results that the pCLE technology can somewhat assist in the differential diagnosis of pleural effusion diseases. Zirlik et al.analyzed pleural effusions from 100 patients ex vivo using pCLE and found that pCLE was able to detect malignant cells in the pleural effusions with a sensitivity of 87% and specificity of 99%, compared to the sensitivity of 80% and specificity of 100% of pleural effusion cytology, showing that pCLE was not inferior to pleural effusion cytology, which is the current “gold standard” (37). A recent prospective (n=15) study by Wijmans et al. on CLE-guided biopsy of malignant pleural mesothelioma evaluated different sampling methods in pCLE-guided biopsy of suspected malignant pleural mesothelioma with specified signs and found that pCLE differentiated malignant mesothelioma from pleural fibrosis with high precision (38). Another focus of our study was to summarize the diagnostic features of pCLE for diagnosing MPE-related diseases by observing and analyzing the pCLE features of MPE. We found during pCLE observation that the pleura of patients with malignant effusion showed a large number of dark black granules of different shapes and sizes, and uneven distribution, either scattered or in clusters, which were considered to be tumor cells. These heterogeneous dark black granular tumor cells were surrounded by significantly twisted blood vessels, and the pleural surface of some patients appeared to have glandular structures, and the glandular cells showed nested or tubular distribution, surrounded by the scattered black granular cell clusters. These features are considered as diagnostic features of pCLE for patients with MPE. These pCLE diagnostic features of MPE summarized in this study are expected to provide a new diagnostic idea for patients with unexplained pleural effusion suspected to be malignant. By reviewing the confocal diagnostic results in conjunction with the final histopathological findings, we identified that the false-positive results (2 cases) were primarily attributable to the overinterpretation of clusters of inflammatory cells. The false-negative result (1 case) was due to a misdiagnosis of an early small tumor focus. All false results were confirmed through repeated histopathological examinations. Therefore, its utility is limited by potential interpretive errors (overreading/underreading), which highlights the need for standardized training and larger cohorts to validate its performance. pCLE can serve as a robust complementary method to existing diagnostic approaches for differentiating BPE from MPE in patients with undetermined etiology. Nevertheless, we still hope that a large number of larger scale multicenter clinical trials will be carried out in the future to validate and summarize the relevant pCLE features of pleural effusion to generate a diagnostic pCLE atlas for pleural effusion. In terms of safety-related results, no adverse events occurred in any of the examined patients, except for the occurrence of skin and scleral icterus associated with NaFl injections, which further confirmed the good security of the technology.

One limitation of this study is the small sample size, leading to a lack of characterization of specific types of benign and malignant pleural effusion on pCLE. Larger, multicenter clinical trials are needed to characterize the pCLE features of different types of benign and malignant pleural effusions to further improve the diagnostic efficacy of this technology. Furthermore, the absence of long-term follow-up limits our understanding of outcomes for patients with negative diagnoses. Similar to the above discussion, as the gold standard of such diagnostic tests, the results of pathologic biopsy are largely influenced by the endoscopic observation site, the physician’s operating habits, and a number of other factors, which are highly random and do not well reflect the efficacy of the diagnostic tests, and therefore need to be corroborated by a long follow-up observational study. This will substantially increase the time cost and financial burden for researchers, but it will be of great benefit to the development of the respiratory medicine field.

In conclusion, pCLE combined with medical thoracoscopy has high diagnostic value for the diagnosis of MPE, and both technologies have their own advantages and complement each other. Medical thoracoscopy piggybacked with pCLE can achieve precise identification of malignant lesions that are difficult to distinguish via regular light microscopy or guide precise biopsy under the thoracoscope, which can greatly improve the accuracy of clinical diagnosis, while reducing the overall diagnostic duration and improving diagnostic efficiency at the same time. Therefore, we believe that pCLE combined with medical thoracoscopy is expected to be more widely used and popularized in the diagnosis of pleural effusion of unknown etiology.

## Data Availability

The original contributions presented in the study are included in the article/supplementary material. Further inquiries can be directed to the corresponding authors.
